# The Cytotoxicity of the Ajoene Analogue BisPMB in WHCO1 Oesophageal Cancer Cells Is Mediated by CHOP/GADD153

**DOI:** 10.3390/molecules22060892

**Published:** 2017-05-28

**Authors:** Vuyolwethu Siyo, Georgia Schäfer, Roger Hunter, Andriy Grafov, Iryna Grafova, Martin Nieger, Arieh A. Katz, M. Iqbal Parker, Catherine H. Kaschula

**Affiliations:** 1 Department of Integrative Biomedical Sciences, University of Cape Town, 7925 Cape Town, South Africa; vuoy.siyo@gmail.com (V.S.); georgia.schafer@uct.ac.za (G.S.); arieh.katz@uct.ac.za (A.A.K.); iqbal.parker@uct.ac.za (M.I.P.); 2 Institute of Infectious Disease and Molecular Medicine, University of Cape Town, 7925 Cape Town, South Africa; 3 Department of Chemistry, University of Cape Town, 7700 Cape Town, South Africa; roger.hunter@uct.ac.za; 4 Department of Chemistry, University of Helsinki, 00014 Helsinki, Finland; andriy.grafov@helsinki.fi (A.G.); iryna.grafova@helsinki.fi (I.G.); martin.nieger@helsinki.fi (M.N.); 5 International Centre for Genetic Engineering and Biotechnology, 7925 Cape Town, South Africa; 6 Department of Chemistry and Polymer Science, Stellenbosch University, 7600 Stellenbosch, South Africa

**Keywords:** ajoene, garlic, cancer prevention, ER stress, unfolded protein response, CHOP/GADD153

## Abstract

Garlic is a food and medicinal plant that has been used in folk medicine since ancient times for its beneficial health effects, which include protection against cancer. Crushed garlic cloves contain an array of small sulfur-rich compounds such as ajoene. Ajoene is able to interfere with biological processes and is cytotoxic to cancer cells in the low micromolar range. BisPMB is a synthetic ajoene analogue that has been shown in our laboratory to have superior cytotoxicity to ajoene. In the current study we have performed a DNA microarray analysis of bisPMB-treated WHCO1 oesophageal cancer cells to identify pathways and processes that are affected by bisPMB. The most significantly enriched biological pathways as assessed by gene ontology, KEGG and ingenuity pathway analysis were those involving protein processing in the endoplasmic reticulum (ER) and the unfolded protein response. In support of these pathways, bisPMB was found to inhibit global protein synthesis and lead to increased levels of ubiquitinated proteins. BisPMB also induced alternate splicing of the transcription factor XBP-1; increased the expression of the ER stress sensor GRP78 and induced expression of the ER stress marker CHOP/GADD153. CHOP expression was found to be central to the cytotoxicity of bisPMB as its silencing with siRNA rendered the cells resistant to bisPMB. The MAPK proteins, JNK and ERK1/2 were activated following bisPMB treatment. However JNK activation was not critical in the cytotoxicity of bisPMB, and ERK1/2 activation was found to play a pro-survival role. Overall the ajoene analogue bisPMB appears to induce cytotoxicity in WHCO1 cells by activating the unfolded protein response through CHOP/GADD153.

## 1. Introduction

**Figure 1:**
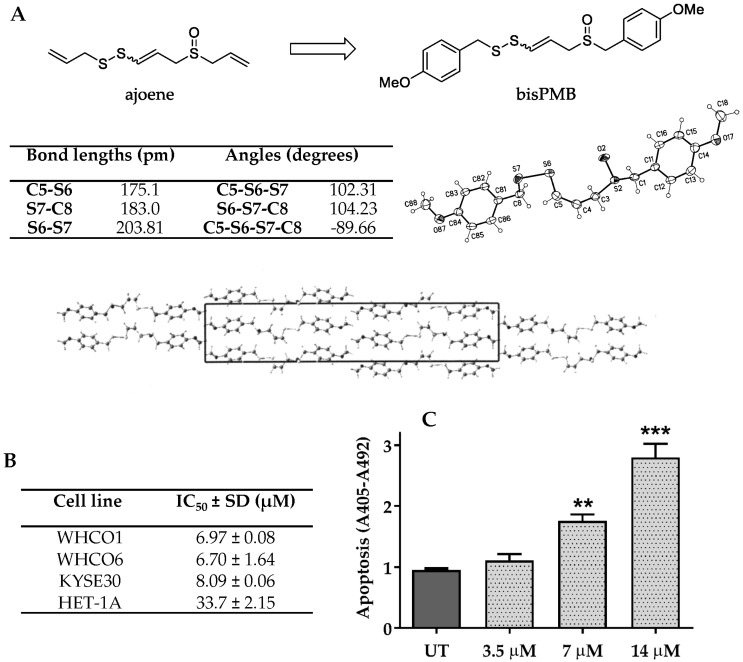
The ajoene analogue bisPMB is cytotoxic to oesophageal cancer cells. (**A**) X-ray crystal structure of bisPMB. After crystallization found to be a pure *Z*-stereoisomer. Table showing structural parameters for =C-S-S-C- moiety in bisPMB. (**B**) Cytotoxicity quantification. Oesophageal cancer cells (WHCO1, WHCO6 and KYSE30) and a non-cancerous cell line (HET-1A) were treated with bisPMB and cytotoxicity was quantitated by the MTT assay. Extent of inhibition is reported as an IC_50_ value ± SD of three independent determinations taken after 24 h treatment. (**C**) Apoptosis quantification. WHCO1 cells were treated with bisPMB at ½ IC_50_; IC_50_ or 2 IC_50_ concentrations and apoptosis was quantified by measuring histone associated DNA fragments in the cytoplasm after treatment. Each bar represents the mean absorbance ± SD of three independent determinations.

Garlic is popular throughout the world for its ability to enhance the flavour of food as well as for its beneficial health effects. There is much epidemiological evidence supporting a link between garlic consumption and reduced cancer risk, particularly involving cancers of the gastrointestinal tract [1,2,3,4,5]. Garlic releases an array of bioactive compounds when the clove is damaged (crushed) in chemical defence against pathogen invasion. Ajoene (see Figure 1A for structure) is a rearrangement product of the primary product allicin, and is cytotoxic to cancer cells in the low micromolar range [6,7,8,9]. Structurally, ajoene has an interesting vinyl disulfide functional group [10] that is rarely found in other natural products. 

Disulfides are common in biological systems where they undergo mixed disulfide exchange reactions with biological thiols, and it is thought that ajoene and related compounds may mimic this reaction. Indeed, a number of garlic polysulfanes have been shown to spontaneously oxidise glutathione to GSS-allyl [11,12]. In vitro experiments have also demonstrated that certain proteins are susceptible to *S*-thiolation by garlic polysulfanes [13,14,15,16,17], and using a tagged ajoene analogue we found that ajoene targets and *S*-thiolates a multitude of proteins in cancer cells [18]. We have also found a correlation between the leaving group p*K*_a_ and cancer cytotoxicity within a library of structurally related disulfides which further supports a thiolysis exchange mechanism driving the cytotoxicity [19]. Ajoene and related compounds are also able to generate reactive oxygen species (ROS), which may contribute to the observed cytotoxic effects [20], although we found that inhibitors of ROS did not abolish the cytotoxicity of garlic related disulfides in WHCO1 oesophageal cancer cells [19]. There is evidence that garlic polysulfanes (S ≥ 3, i.e., the garlic compound diallyl trisulfide) release H_2_S through thiol-disulfide exchange following allyl perthiol reduction with GSH, and this reaction may also contribute to the cytotoxic mechanism of trisulfides in cancer cells [21].

Apoptotic signalling by garlic polysulfanes has been shown to proceed via the intrinsic pathway through the mitochondrial-dependent caspase cascade involving activation of caspase 3 and cleavage of the anti-apoptotic protein Bcl-2 [22,23]. Apoptotic signaling may be triggered upstream by the mitogen-activated kinase (MAPK) pathways. For example, ajoene-induced cancer cell death activates the c-Jun NH_2_-terminal kinase (JNK), p38 and extracellular signal-regulated kinases (ERK)1/2 as well as the survival kinase Akt [24,25]. Other garlic polysulfanes namely *S*-alylmercaptocysteine (SAMC) [26], diallyl disulfide (DADS) [27], diallyl trisulfide (DATS) [28] and dialyl tetrasulfide (DAS4) [29] have all been shown to also activate the JNK pathway in a range of different cancer cell lines. 

There is a growing body of evidence that ER stress may play a role in the cytotoxicity of garlic polysulfanes in cancer cells. Using a fluorescently-labelled ajoene analogue, we found that ajoene targets and accumulates within the endoplasmic reticulum (ER) of cancer cells [18]. Ajoene was found to accumulate in this organelle through a trapping mechanism involving mixed disulfide formation with exposed cysteine residues on newly synthesized proteins. This leads to an accumulation of misfolded proteins that activate the unfolded protein response (UPR) [18]. We observed increased levels of the GRP78 protein in MDA-MB-231 breast cancer cells [18], which is in agreement with findings by Wang et al. who observed that the ER stress markers CHOP and GRP78 are induced in BCC cells following treatment with DATS [30]. Additionally, DAS4 is reported to induce activation of the ER stress protein EIF2α as well as the expression of the ER stress receptor ATF4 in HCT116 human colon cancer cells [31,32]. It has long been known that garlic polysulfanes influence calcium homeostasis in cultured cancer cells [31,33,34,35,36,37,38,39,40], which is an early indicator of ER stress.

The compound bisPMB, has previously emerged from our lab as a synthetic ajoene analogue with enhanced cytotoxicity against a range of cancer cell lines and a degree of selectivity for cancer cells over non-cancerous cells [9,41]. Structurally, bisPMB retains the central vinyl disulfide/sulfoxide core of ajoene and the allyl side groups have been substituted for paramethoxybenzyl groups [41]. In the current study we have investigated the transcriptional changes and biological pathways that are enriched and activated by bisPMB in WHCO1 oesophageal cancer cells.

## 2. Results

### 2.1. The Ajoene Analogue BisPMB Is Cytotoxic to Oesophageal Cancer Cells

The compound bisPMB has previously been developed in our laboratory [9,41,42] as a promising ajoene analogue that displays up to twelve-fold enhanced cytotoxicity against a range of cancer cell lines, as well as a degree of selectivity (2–3 fold) for cancer cells over their non-cancerous counterparts. Synthetically, bisPMB is purified by column chromatography in solution as a mixture of inseparable *E*/*Z*-stereoisomers which are assigned on the basis of their ^1^H-NMR vinyl coupling constants; approximately 15 Hz for the *E*-isomer and 10 Hz for the *Z*-isomer. We found that crystallisation enriches only for the *Z*-isomer as confirmed by ^1^H-NMR spectroscopy and X-ray crystallography. The X-ray crystal structure of bisPMB is shown in Figure 1A. The distinguishing feature of the molecule is the asymmetrically substituted disulfide bridge =C-S-S-C-, for which the bond lengths, angles, and the dihedral angle are presented in Figure 1A.

Since bisPMB previously showed good activity against the WHCO1 oesophageal cancer cell line [9], we decided to investigate its cytotoxic mechanism in more detail. BisPMB was found to inhibit proliferation of the three oesophageal cancer cell lines WHCO1, WHCO6 and KYSE30 with very similar 24 h cytotoxicity IC_50_’s in the range of 6–8 μM (see Figure 1B). The WHCO1 and WHCO6 cell lines are derived from biopsies of South African patients [43], while the KYSE30 cell line is derived from a biopsy of a Japanese male [44]. A 5-fold selectivity was observed for the cancer cells over the non-cancerous Het-1A epithelial cell line (see Figure 1B). The reported 24 h IC_50_ values for cisplatin, one of the oesophageal cancer chemotherapeutic drugs is in the range of 38 μM to 56 μM [45]; thus the range of 6–8 μM for bisPMB in addition to its 5-fold selectivity for cancer cells places it in a promising therapeutic space. 

Apoptosis is widely implicated in the cytotoxicity of cancer chemotherapeutic drugs. By monitoring the increase in histone-associated DNA fragments in the cytoplasm of WHCO1 cells after 24 h treatment, we found that bisPMB induces apoptosis in a dose-dependent manner (see Figure 1C). The cell viability and apoptosis quantification appears to correlate well as apoptosis was only observed at concentrations of the IC_50_ value (7 µM) and above. No apoptosis was observed at the ½ IC_50_ (3.5 µM) treatment concentration. 

### 2.2. Transcriptional Profiling of BisPMB-Treated WHCO1 Cells

Recent evidence from our laboratory has shown that ajoene accumulates in the endoplasmic reticulum of MDA-MB-231 breast cancer cells by *S*-thiolating newly synthesized proteins [18]. We further showed that thiolysis exchange with ajoene leads to an accumulation of misfolded proteins which are known to be toxic to cells as they form insoluble aggregates and activate the unfolded protein response [46,47]. In order to further investigate and characterize the cytotoxicity of bisPMB, we performed DNA microarray analysis using the Affymetrix human gene ST 2.0 array to compare gene expression profiles in untreated and in bisPMB-treated WHCO1 cells. We chose to treat the WHCO1 cells with the ½ IC_50_ concentration of bisPMB for 24 h as this was shown to be non-cytotoxic and would enable us to investigate early transcriptional changes prior to apoptosis. Total RNA was extracted, reverse-transcribed into cDNA and hybridized into the Affymetrix gene ST 2.0 array GeneChip (Thermo Fisher Scientific, Waltham, MA, USA). The array was then scanned and the data was analysed using the Partek Genomics Suite software (version 6.6). 

**Figure 2:**
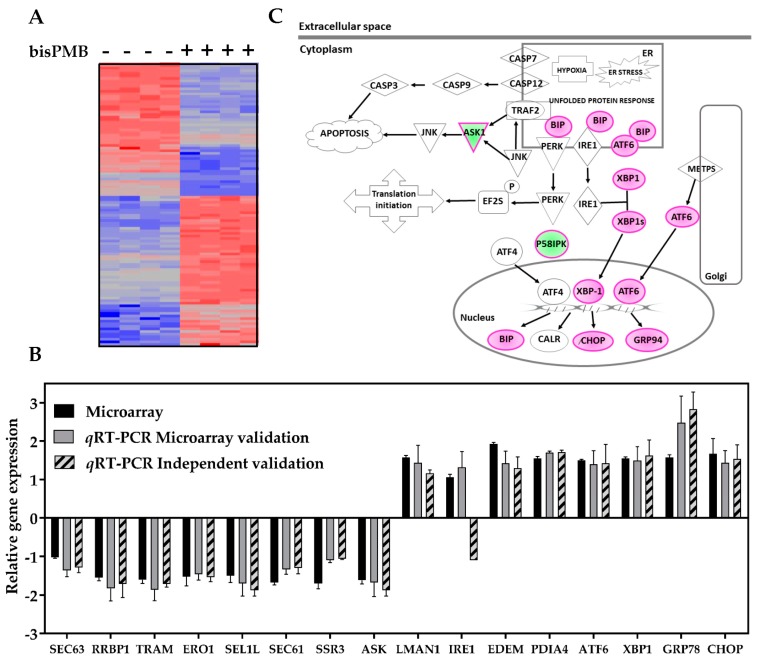
Transcriptional profiling of WHCO1 oesophageal cancer cells treated with bisPMB. (**A**) Microarray hierarchical clustering. The hierarchical clustering of genes from the Affymetrix Gene Chip Human Gene 2.0 ST Array consisting of four experimental replicates of control and bisPMB-treated WHCO1 cells. Each gene is represented by a column, and each sample is represented by a row. Expression changes: unchanged (grey), up-regulated (red), down-regulated (blue). (**B**) Microarray validation. The fold change of 16 DEGs obtained from the microarray (black) were validated by reanalysing the same microarray mRNA by *q*RT-PCR (grey); and independently by extracting new mRNA from an independent experiment and analysing by *q*RT-PCR (hashed). All data was normalized relative to GAPDH. (**C**) Gene network of the ER stress pathway. The DEGs from the microarray are highlighted in green (down-regulated) or red (up-regulated). The cellular compartments are labelled inside the shape demarcating them.

Principle component analysis and hierarchical clustering (see Figure 2A) demonstrated that the biological replicates clustered reproducibly within each sample group. Two-way ANOVA was used to select the differentially expressed genes (DEGs) between the two samples, and multiple testing of the *p*-values was conducted by Bonferroni testing to calculate the false discovery rate (FDR) [48]. The cut-off for the DEGs between treated and untreated samples was set at 1.5 fold, with a statistically significant FDR of α ≤ 0.05. A total of 488 DEGs out of 24,000 total genes were found to be statistically deregulated in the bisPMB treated sample.

#### 2.2.1. Validation of Selected Microarray DEGs 

The microarray results were validated by *q*RT-PCR on the same RNA samples used for the microarray, and also independently by extracting new RNA from two independent repeats. The validation was conducted on 16 DEGs which were chosen based on their involvement in protein processing in the ER (see Table S1 of the Supplementary section for details of the genes and PCR primers used for each gene). The *q*RT-PCR results for all the DEGs were in agreement with the microarray data (with the exception of the independent experiment targeting IRE1) (see Figure 2B). Thus we were satisfied that the RNA sent for microarray analysis gave good quality results and that the fold changes observed were reproducible and indicative of the transcriptional changes in WHCO1 cells. A list of the top 16 DEGs most deregulated by bisPMB is given in Table S2 (Supplementary section). These included genes which are implicated in inflammation and inflammatory responses to cancer (PTX3, IL8, TNFSF10, SERPINB13 and ANKRD1) and those involved in cell adhesion, cell migration and ECM degradation of importance in metastatic cancer (MMP12, MMP1 and ADAM19).

#### 2.2.2. Gene Ontology Analysis

In order to identify the most prominent biological processes enriched by bisPMB, gene ontology (GO) analysis was performed using WebGestalt2 2013 WEB-based Gene SeT AnaLysis Toolkit. According to ancestral categories, the GO biological processes most affected by bisPMB included: metabolic process, biological process and response to stimuli (see Figure S1). Higher resolution into specific biological processes was achieved using a directed acyclic graph (DAG). The DAG illustrated that the “ER nucleus signalling pathway” followed by “response to topologically incorrect protein” and “response to unfolded protein” were the most significant (see Table S3.1). 

In order to investigate the cellular components relevant to the bisPMB DEGs, bar chart and directed acyclic graphs were constructed. The top cellular components affected in bisPMB-treated WHCO1 cells included the categories: membrane, nucleus, membrane enclosed lumen and endomembrane system (see Figure S2). Higher resolution into specific biological processes was achieved using a directed acyclic graph (DAG). The DAG illustrated that the “nuclear outer membrane-endoplasmic reticulum membrane network” was the most significant with an adjP/FDR value of 1.58 × 10^−7^ containing 34 genes (see Table S3.2). This finding complements the findings of the enriched biological processes as the UPR is associated with genes that target the ER membrane and lumen. In order to gain more information into the underlying molecular events involved, a pathway analysis was conducted.

#### 2.2.3. Pathway Analysis

Examination of the molecular pathways activated by bisPMB was conducted using the KEGG pathway network module and the ingenuity pathway analysis (IPA). Numerous KEGG pathways were found to contain bisPMB DEGs (see Figure S3). The three categories with the largest number of bisPMB DEGs included pathways in cancer (25 genes), protein processing in the endoplasmic reticulum (21 genes) and the MAPK signalling pathway (17 genes). Further resolution of the KEGG pathway network revealed enrichment of the ER protein processing pathways, which include: UPR, endoplasmic reticulum associated degradation (ERAD) and protein translocation pathways (see Figure S4). The specific DEGs in the UPR pathways included ATF6, IRE-1, XBP-1, GRP78/BIP and CHOP, all of which were up-regulated. The ERAD pathway included DEGs such as EDEM, Ubc6 and VIMP. Moreover, the majority of the DEGs represented in the protein translocation pathways were down-regulated and these included Sec63, TRAM, SEC24D and SSR3/TRAP. These findings suggest that WHCO1 cells respond to bisPMB by up-regulating genes involved in enhancing the UPR and ERAD; and down-regulating genes that play a role in the import of proteins to the ER involved in the protein translocation pathways to the ER. Canonical pathway analysis generated using the ingenuity pathway analysis (IPA) software found that the ER stress pathway was the most significantly enriched, with a total of 9.5% down-regulated and 23.8% up-regulated DEGs. A number of pathways implicated in cancer were also enriched. Interestingly, pathways involved in cholesterol biosynthesis were enriched by bisPMB, which supports literature reports that dietary garlic lowers cholesterol levels [49,50].

#### 2.2.4. Network Analysis

The gene network underlying the ER stress pathway was generated using IPA. There are three ER transmembrane proteins IRE-1, PERK and ATF6, which are the sensors and transducers of the UPR signalling pathway. The Gene Network of the ER stress pathway showed an up-regulation of ATF6, with no change in the transcriptional levels of IRE1, PERK and ATF4 (see Figure 2C). Additionally, the molecular chaperones GRP78/BIP and GRP94, and the transcription factors CHOP and XBP1 were up-regulated while ASK and the PERK inhibitor p58i were down-regulated. The IPA ranks networks according to the number of DEGs it contains and the number of connections arising between the genes in each network. We observed that the most highly ranked molecular network in the bisPMB sample comprised 34 DEGs, containing ER stress genes ATF6, XBP1 and GRP78/BiP (see Figure S5). In addition, the DEG with the highest number of connections to other genes in this network is XBP-1. This suggests that XBP-1 may be the most biologically relevant gene in this network, as it influenced a number of genes that are involved in various other cellular functions through network connections. Overall the findings suggest that bisPMB may act primarily by triggering the UPR and ER stress pathways. Furthermore the molecular gene network analysis showed that the XBP-1 gene is central to these intracellular functions by regulating the expression of the highest number of DEGs. 

### 2.3. BisPMB Activates the Unfolded Protein Response

**Figure 3:**
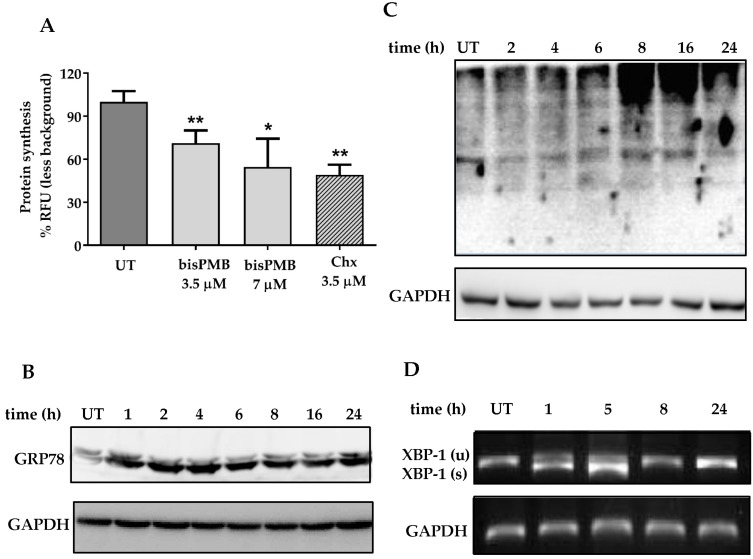
bisPMB activates the unfolded protein response in WHCO1 cells. (**A**) Protein synthesis quantification. WHCO1 cells were treated with bisPMB (IC_50_ concentration) for 24 h and protein synthesis was quantified fluorometrically using the Click-it HPG Alexa Fluor Protein Synthesis Assay kit. (**B**) WHCO1 cells were incubated with bisPMB (½ IC_50_ concentration) for up to 24 h. Total cell lysate was extracted and analysed for GRP-78 expression by immunoblot probed with anti-GRP78 and anti-GAPDH primary antibodies. (**C**) WHCO1 cells were incubated with bisPMB (½ IC_50_ concentration) for up to 24 h. Total cell lysate was extracted and examined for total ubiquitination by immunoblot using anti-ubiquitin and anti-GAPDH primary antibodies. (**D**) Time dependent splicing of XBP1. mRNA was extracted from WHCO1 cells treated with bisPMB (IC_50_ concentration) for up to 24 h and subjected to *q*RT-PCR. The XBP-1 spliced (s) and unspliced (u) PCR products were separated on an agarose gel. Immunoblots and gels shown are representative of two independent experiments. The graphs are an average of three independent experiments. Student *t*-test indicates * *p*-value < 0.05; ** *p*-value < 0.01.

The findings of the microarray and subsequent *in silico* analysis support previous findings from our laboratory in which a fluorescently-labelled ajoene was found to localise in the ER of cancer cells. We further found that ajoene targets and *S*-thiolates cysteine residues of newly synthesized proteins, which leads to an accumulation of misfolded proteins that triggers the unfolded protein response. The unfolded protein response is an evolutionary conserved response to an accumulation of misfolded proteins [46,47]. The role of UPR is to adapt and reestablish normal ER function. These adaptive mechanisms involve transcriptional programs that (1) inhibit further protein synthesis and enhance protein folding capacity, as well as, (2) promote ER-associated degradation (ERAD) [51,52] to remove incorrectly folded proteins. In order to probe whether bisPMB activates the unfolded protein response, protein synthesis was measured using the Click-it Protein Synthesis Assay. This assay tracks the incorporation of a labelled methionine analogue into proteins during active protein synthesis. BisPMB was found to inhibit protein synthesis by 1.6 and 2 fold when treated at ½ IC_50_ (3.5 µM) and at IC_50_ (7 µM) concentrations, respectively (see Figure 3A). As a positive control, treatment with the known protein synthesis inhibitor cycloheximide (3.5 µM) for 24 h led to a 2-fold reduction in protein synthesis. From this data it appears that bisPMB attenuates protein synthesis in WHCO1 cells. 

In an attempt to enhance the protein folding capacity in the ER, one arm of the UPR is to stimulate increased production of chaperones. GRP78 is a Ca^2+^ dependent chaperone that is involved in protein folding and is released from the ER transmembrane signaling proteins when the levels of misfolded proteins increase [53]. We have previously shown that *Z*-ajoene increases the expression of GRP78 in MDA-MB-231 breast cancer cells [18]. In the current study, we found that bisPMB increases the expression of GRP78 in WHCO1 cells, which was observed from the earliest time point measured (1 h) and sustained through to 24 h (see Figure 3B). 

ER-associated degradation is activated in response to an accumulation of misfolded proteins. The ubiquitin proteasome system forms an integral part of ERAD where misfolded proteins are ubiquitinated and targeted for degradation. In WHCO1 cells, bisPMB activated ERAD by increasing the global levels of ubiquitinated proteins by 8 h after treatment (see Figure 3C). A similar effect has previously been observed in MDA-MB-231 breast cancer cells treated with *Z*-ajoene [18].

The microarray data revealed that XBP-1 is the most biologically relevant gene in the ER stress gene network. There are three receptor pathways that lead to the ER stress response and XBP-1 forms part of the IRE-1 pathway. During activation, XBP-1 mRNA is spliced and translated into a transcription factor that induces the expression of ER stress genes by binding to the ER stress response element (ERSE). RNA isolated from untreated and bisPMB treated cells were subjected to qRT-PCR using primers able to distinguish between the two alternately spliced XBP-1 RNAs to generate PCR products of either 283 or 257 bp. The alternately spliced RNA was present between 1–5 h after treatment but not at the later time points (8–24 h) (see Figure 3D). Taken together with the findings of the microarray data, it appears that bisPMB activates the IRE-1/XBP-1 ER stress pathway and that XBP-1 splicing may be an early response to bisPMB in WHCO1 cells. 

### 2.4. CHOP/GADD153 Is a Central Regulator of BisPMB-Induced Cytotoxicity

**Figure 4:**
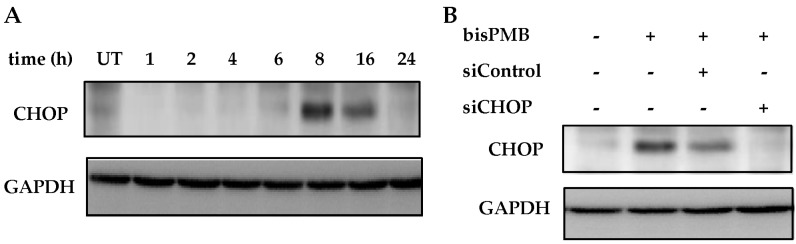

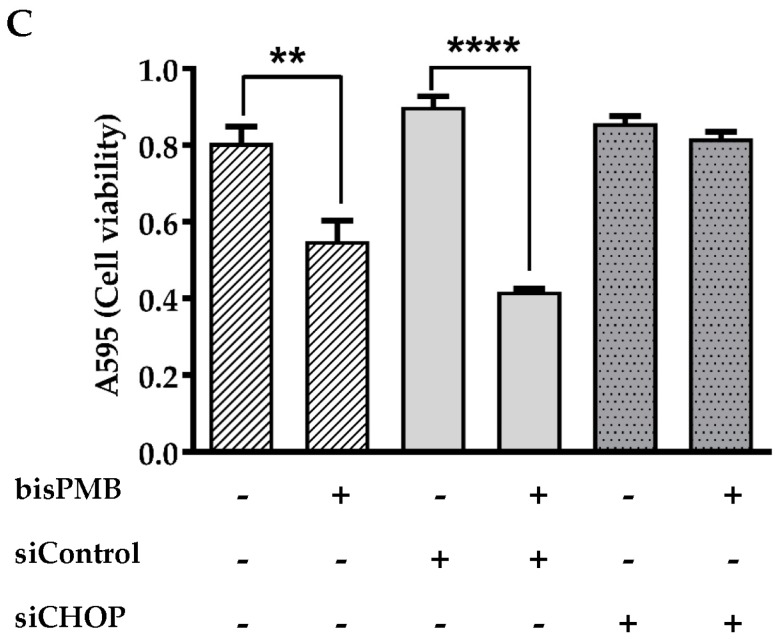
CHOP knock down reverses bisPMB cytotoxicity in WHCO1 cells. (**A**) Time-dependent CHOP expression. WHCO1 cells were incubated with bisPMB (IC_50_ concentration) for 24 h. Total cell lysate was then extracted and examined for time-dependent CHOP protein expression by immunoblot probed with anti-CHOP and anti-GAPDH primary antibodies. (**B**) Knock-down of CHOP. WHCO1 cells were pre-incubated with siRNA-CHOP or vehicle for 6 h followed by addition of bisPMB (IC_50_ concentration) for 24 h. Total cell lysate was extracted and examined for CHOP protein expression by immunoblot probed with anti-CHOP and anti-GAPDH primary antibodies. (**C**) Cell viability quantification. WHCO1 cells were pre-incubated with siRNA-CHOP or vehicle for 6 h followed by addition of bisPMB (IC_50_ concentration) for 24 h. Cell viability was then quantified by the MTT assay. Each column represents an average of three technical replicates and the graphs are representative of three independent experiments. Student *t*-test indicates ** *p*-value < 0.01; **** *p*-value < 0.001.

The C/EBP homologous protein CHOP, is a bZIP-containing transcription factor and a member of the CCAAT/enhancer binding protein (C/EBP) family, also known as DNA-damage-inducible gene 153 (GADD153). During prolonged ER stress, CHOP is one of the most highly up-regulated genes [54,55]. The chop gene promoter has binding sites for all the major inducers of UPR, including ATF4, ATF6 and XBP-1. Being a transcription factor, CHOP does not induce apoptosis directly, but rather up-regulates target genes such as GADD34 and ERO1α which promote ER stress conditions. CHOP also downregulates anti-apoptotic proteins (for example Bcl-2) that promote apoptosis. CHOP was not detected in untreated WHCO1 cells although it was significantly induced by bisPMB (see Figure 4A). To investigate whether CHOP plays a role in the cytotoxicity of bisPMB, cell viability was quantitated under conditions where CHOP had been silenced using siRNA. Immunoblot analysis showed that the CHOP protein was successfully silenced by siRNA-CHOP but was not affected in the siRNA-Control cells (see Figure 4B). WHCO1 cells were then pre-treated with siRNA-CHOP or siRNA-Control followed by the addition of the IC_50_ concentration of bisPMB for 24 h; after which the cell viability was quantitated by the MTT assay. As expected, the IC_50_ concentration of bisPMB reduced WHCO1 cell viability to half the initial value (see Figure 4C). The siRNA-Control cells retained their sensitivity to bisPMB; however those cells in which CHOP had been silenced, were resistant to the cytotoxic effects of bisPMB. These findings demonstrate that CHOP/GADD153 plays a key and central role in the cytotoxicity of bisPMB in WHCO1 cells.

### 2.5. The Role of the MAPK Signaling Pathways in BisPMB-Induced Cytotoxicity

**Figure 5:**
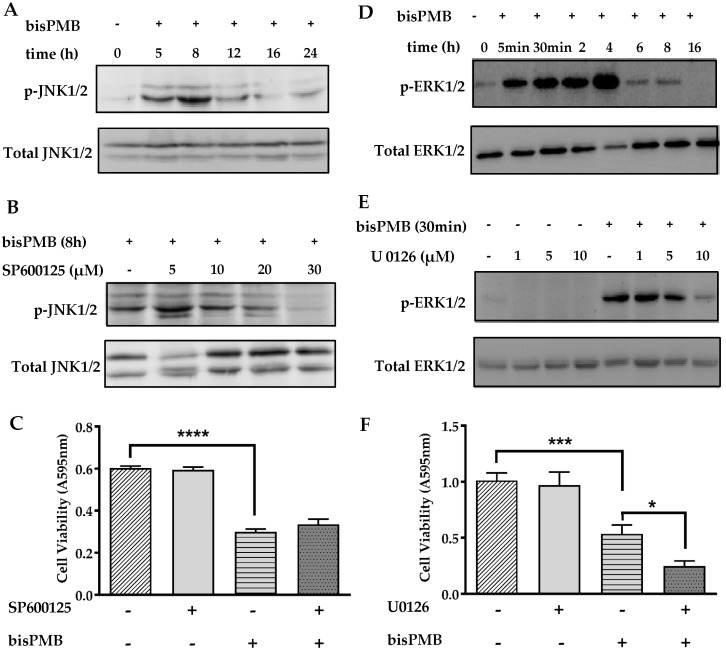
BisPMB induces time dependent MAPK activation in WHCO1 cells. (**A**) WHCO1 cells were treated with bisPMB (IC_50_ concentration) for up to 24 h. The cell lysate was collected and analysed by immunoblot probed with anti-phosphorylated JNK1/2 and total anti-JNK1/2 primary antibodies. (**B**) WHCO1 cells were pre-treated with SP600125 at the indicated concentration for 30 min followed by the addition of bisPMB (IC_50_ concentration) for 8 h. The cell lysate was collected and analysed by immunoblot probed with anti-phosphorylated JNK1/2 and total anti-JNK1/2 primary antibodies. (**C**) WHCO1 cells were pre-treated with 30 µM SP600125 for 30 min followed by the addition of bisPMB (IC_50_ concentration) for 24 h. Cell viability was then quantified by the MTT cytotoxicity assay. (**D**) WHCO1 cells were treated with bisPMB (IC_50_ concentration) for up to 16 h. The cell lysate was collected and analysed by immunoblot probed with anti-phosphorylated ERK1/2 and total anti-ERK2 primary antibodies. (**E**) WHCO1 cells were pre-treated with U0126 at the indicated concentration for 2 h followed by the addition of bisPMB (IC_50_ concentration) for 30 min. The cell lysate was collected and analysed by immunoblot probed with anti-phosphorylated ERK1/2 and total anti-ERK2 primary antibodies. (**F**) WHCO1 cells were pre-treated with 10 µM U0126 for 2 h followed by bisPMB (IC_50_ concentration) for 24 h. Cell viability was then quantified by the MTT cytotoxicity assay. Immunoblots shown are representative of two independent experiments. Each bar represents the average absorbance reading from three technical replicates and the experiment was performed in dependently in triplicate. Student *t*-test indicates * *p*-value < 0.05; *** *p*-value < 0.005; **** *p*-value < 0.001.

There is evidence that UPR-induced apoptosis is linked to MAPK signaling [56]. The JNK MAPK proteins belong to the stress activated protein kinase (SAPK) family of proteins that are activated by stress stimuli, such as chemotherapeutic drugs, and are associated with apoptosis [57,58]. In contrast, the MEK/ERK pathway is a pro-survival pathway which has been implicated in apoptosis suppression [59]. Some of the garlic polysulfanes are reported to enhance the activation of MAPK pathways in vitro. For example DADS, DATS and ajoene have been shown to activate the JNK, p38 and MEK/ERK signalling in various cancer cell lines [24,26,27,28,29]. In our microarray study (see Figure S3), transcriptional changes associated with the MAPK signalling pathways were found to be enriched. We therefore investigated whether bisPMB induces activation of the MAPK signalling pathways in WHCO1 cells. We observed that bisPMB increased JNK1/2 phosphorylation in WHCO1 cells in a time dependent manner with maximal activation between 5–8 h (see Figure 5A). The JNK1/2 inhibitor SP600125 inhibited JNK phosphorylation in a concentration dependent manner and a concentration of 30 μM was selected for further experiments (see Figure 5B). WHCO1 cells were then pretreated with the inhibitor followed by IC_50_ treatment with bisPMB for 24 h. At the end of the experiment, cell viability was quantified by the MTT assay (see Figure 5C). As expected, bisPMB reduced the viable cells to half the initial value, while the inhibitor alone, or in combination with bisPMB had no effect on the cytotoxicity of bisPMB. These results demonstrate that JNK phosphorylation is not critical in the cytotoxicity of bisPMB. 

The MEK/ERK pathway is a survival MAPK pathway that is activated by a number of garlic-derived compounds including ajoene [24]. The activation of this pathway is usually evaluated by assessment of phosphorylated ERK1/2. BisPMB at the IC_50_ concentration enhanced the ERK1/2 phosphorylation in a time-dependent manner (see Figure 5D), with pronounced phosphorylation within 5 min and reaching a maximum at 4 h. These levels, however, declined from 6 h, with complete reversal of activation by 16 h, implying a transient, early activation of ERK1/2 in WHCO1 cells. In order to determine the role of MEK/ERK signalling in the cytotoxicity of bisPMB, the MEK1/2 inhibitor, U0126 was used. The reduction in ERK1/2 phosphorylation was clearly visible in bisPMB-exposed cells treated with 10 µM U0126 (see Figure 5E). WHCO1 cells were then treated with this inhibitor in combination with bisPMB (IC_50_ concentration), and cell viability was measured at the end of the experiment. WHCO1 cells treated with bisPMB alone exhibited a 50% reduction in viable cells. The inhibitor U0126 had no effect on WHCO1 cell viability, although treatment with the combination caused the cells to become more sensitised to the effects of bisPMB (see Figure 5F). These results suggest that the activation of the MEK/ERK pathway in WHCO1 cells plays a pro-survival role, with inhibition of ERK1/2 seemingly sensitising the cells to bisPMB.

## 3. Discussion

Identification of novel intracellular pathways is important in designing drug combinations for cancer therapy, as drug synergism is often attained by drugs that target different pathways [60]. Using tagging technology, we recently showed that ajoene preferentially targets and accumulates in the ER of cells, where it interferes with protein folding and activates the unfolded protein response [18]. In addition, the cytotoxicity of garlic-related disulfides is dependent on *S*-thiolation which in turn is driven by the stability of the leaving group in a thiolysis exchange reaction [19]. Taken together our findings support a chemical mechanism that is dependent on thiolysis exchange with proteins in the ER. To our knowledge this is a novel mechanism amongst currently known cancer cytotoxic agents. ER-stress inducing agents exist, such as thapsigargin and tunicamycin [61], however, they do not target protein processing in the ER.

An interesting particularity of ajoene, and bisPMB as the ajoene family member, is the presence of an asymmetrically substituted =C-S-S-C- bridge. In this paper we report for the first time the crystal structure of bisPMB. Previously, only ajoene cyclodextrin complexes were investigated [62]. As shown in Figure 1A, the values of the corresponding C-S and S-S bond lengths are within the typical range for organic disulfides, where the C-S bond is slightly shorter on the vinyl-S side. This is indicative of resonance stabilization of a vinyl-S lone pair into the double bond, which renders that sulfur slightly δ+ in the resonance structure. In turn, this supports the observed regioselectivity of thiolysis, in which attack at the non-vinyl sulfur is preferred in order to restore change at the vinyl one. It also accommodates the greater reactivity of the vinyl sulfide grouping over that of the saturated disulfide in that the polarization makes for an enhanced electrophilicity on the non-vinyl S of the disulfide as well as endowing the vinylthio moiety with a superior leaving ability compared to that of the saturated disulfide case. The S-S bond length in turn is slightly shorter than that reported for L-cysteine × 2HBr (202.4 pm) [63]. The dihedral angle =C-S-S-C- of −89.66° ideally corresponds to the right angle value expected for the minimum energy configuration owing to the repulsion of lone-pair electrons on sulfur atoms. It also shows that the S-S bonding is the main factor determining the dihedral value, rather than the intermolecular interactions within the unit cell in a crystalline phase [64]. 

BisPMB is a synthetic analogue of ajoene with superior cytotoxicity, as well as some selectivity for cancer over non-cancerous cells, especially WHCO1 oesophageal cancer cells. In view of the apparent ER targeting of ajoene as well as the enhanced cytotoxicity of bisPMB, we performed a transcriptional gene microarray analysis on bisPMB-treated WHCO1 cells. For this, the cells were treated with a non-cytotoxic (½ IC_50_) concentration of bisPMB to profile the early transcriptional changes that occur. Gene ontology and pathway analysis of the significantly deregulated genes revealed that pathways in cancer, protein processing in the ER and the MAPK signalling pathways were the most significantly enriched by bisPMB. Canonical pathway analysis generated by ingenuity pathway analysis found that the ER stress and UPR pathways were the most significantly enriched.

Misfolded proteins are estimated to comprise approximately 30% of newly synthesized proteins in healthy cells [65], resulting in the need for their removal, which is carried out by ERAD [51]. Elevated levels of misfolded proteins are toxic to cells and trigger the unfolded protein response [47,66], which if not corrected, induces apoptosis. The role of UPR is to adapt and reestablish normal ER function. These adaptive mechanisms involve transcriptional programs that inhibit further protein synthesis and enhance protein folding capacity, and promote ER-associated degradation of incorrectly folded proteins [51,52]. In agreement with an activation of the unfolded protein response, we found bisPMB attenuated global protein synthesis with a concomitant increase in ubiquitinated proteins in response to an increase in misfolded proteins. BisPMB also increased the expression of GRP78 an ER-resident protein that is released from the ER stress receptors when levels of misfolded proteins increase. There are three ER transmembrane proteins IRE-1, PERK and ATF6, which are sensors and transducers of the UPR signalling pathway. From the microarray, we found that XBP-1 is the most biologically relevant gene in the ER stress gene network that forms part of the IRE-1 pathway. We found that bisPMB causes the splicing of XBP-1. Taken together, our data shows that bisPMB activates UPR and that the IRE-1/XBP-1 pathway is implicated. Prolonged UPR triggers ER stress-induced apoptosis, that is associated with the induction of CHOP/GADD153 [55,67]. We observed a time dependent induction of CHOP/GADD153, that peaked between 8 and 16 h after treatment with bisPMB. Furthermore siRNA-CHOP rendered the cells resistant to the cytotoxic effects of bisPMB. Therefore CHOP/GADD153 appears to be the central regulator in the cytotoxicity of bisPMB in WHCO1 cells. Our findings therefore appear to strongly support the hypothesis that bisPMB-induced ER stress increases CHOP expression which is central to its cytotoxicity in WHCO1 cells. The signalling pathway leading to CHOP expression could proceed through PERK–eIF2α–ATF4–CHOP or IRE1–TRAF2–ASK1–JNK or IRE1-XBP1-CHOP or ATF6-CHOP although XBP-1 was found to be the most biologically relevant gene with the highest number of connections to other genes in the ER stress gene network implying that the IRE1 pathway may be important. Suppression of the CCAAT/enhancer binding protein (C/EBP) transcriptional activity could lead to mitochondrial dysfunction [68,69] and the observed apoptosis in WHCO1 cells.

UPR pathways have been implicated in crosstalk with the MAPK signalling pathways [56], and our microarray pathway analysis found the MAPK pathways to be significantly enriched. In support of this, garlic polysulfanes are known to activate the MAPK pathways [24,26,27,28,29]. Inhibition of JNK activation was, however, found to have no effect on bisPMB-induced cytotoxicity, implying that JNK activation is not a critical regulator in the cytotoxicity of bisPMB. Our finding is supported by that of Antlsperger et al. in which inhibition of JNK with SP600125 did not alter *Z*-ajoene-induced apoptosis in HL-60 leukemia cells [24]. 

The MAPK family member ERK1/2 was also activated by bisPMB, which is in agreement with that reported for *Z*-ajoene in human leukemia HL-60 cells [24] and human glioblastoma multiform cancer stem cells [25]. Inhibition of the ERK signaling pathway with U0126, however, sensitised the cells to bisPMB implying that this pathway might play a pro-survival role. This finding is not surprising as the MEK/ERK pathway is known to promote cell survival, differentiation and proliferation [70] and chronic ER stress is reported to promote tumour progression [71]. The MEK/ERK pathway is also commonly implicated in chemotherapeutic resistance [72].

Little is known about the bioavailability and stability of ajoene and related analogues in vivo and this aspect would need to be investigated to ascertain the feasibility of an ajoene-based cancer therapeutic. The ajoene analogue bisPMB appears an attractive target for further cancer therapeutic development. It is cytotoxic to cancer cells in the low micromolar range and appears to have some selectivity for cancer over non-cancerous cells. Importantly it appears to have a novel intracellular target that involves ER targeting and activation of the unfolded protein response through the transcription factor CHOP/GADD153. The ER stress pathway has previously been identified as an attractive target for cancer therapy [73] as manipulation of ER stress has been shown to synergistically enhance the efficacy of chemotherapeutic drugs. 

## 4. Materials and Methods 

### 4.1. X-ray Crystallography

BisPMB was synthesized according to our synthetic route to ajoene analogues and its characterization is reported elsewhere [41,42]. The single-crystal X-ray diffraction study was carried out on a Nonius Kappa CCD diffractometer (Bruker, Billerica, MA, USA) equipped with a CCD detector at 123(2) K using Mo-Kα radiation (*λ* = 0.71073 Å). Direct methods were used for structure solution and refinement was carried out using SHELXL97 (full-matrix least-squares on *F*^2^) [74]. Hydrogen atoms were localized by difference electron density determination and refined using a riding model. A semi-empirical absorption correction was applied. bisPMB: Colourless crystals, C_19_H_2_O_3_S_3_, *M*_r_ = 394.55, crystal size 0.30 × 0.12 × 0.06 mm, monoclinic, space group *P*2_1_/n (No. 14), *a* = 5.152(1) Å, *b* = 8.525(2) Å, *c* = 43.352(8) Å, *β* = 90.69(2)°, *V* = 1903.9(7) Å^3^, *Z* = 4, *ρ* = 1.376 Mg/m^−3^, *µ*(Mo-K_α_) = 0.405 mm^−1^, *F*(000) = 832, *2θ*_max_ = 55.0°, 18006 reflections, of which 4191 were independent (*R*_int_ = 0.058), 228 parameters, *R*_1_ = 0.066 (for 3324 I > 2σ(I)), w*R*_2_ = 0.153 (all data), *S* = 1.20, largest diff. peak/hole = 0.500/−0.476 e Å^−3^. CCDC 1535128 (bisPMB) contains the supplementary crystallographic data for this paper. These data can be obtained free of charge from The Cambridge Crystallographic Data Centre via www.ccdc.cam.ac.uk/data_request/cif. 

### 4.2. Cell Lines and Treatments

The oesophageal cancer cell lines WHCO1 and WHCO6 were derived from biopsies of oesophageal squamous cell carcinomas from South African patients [43]. The KYSE30 cell line was derived from a middle intra-thoracic oesophagus of a 64 year old Japanese man [44]. Het-1A cell line is an oesophageal epithelial cell-line derived from a 25-year-old black male, which has been immortalized with the SV40 Large T antigen (ATCC CRL-2692). The cells were cultured in Dulbecco’s Modified Eagle medium (Gibco^®^, ThermoFisher Scientific, Waltham, MA, USA) supplemented with 10% foetal bovine serum (FBS) (HyClone^™^, GE Healthcare Life Sciences, Chicago, IL, USA) and 100 µg/mL penicillin and 100 µg/mL streptomycin (Biochrom, Cambridge, UK). Cells were incubated in a humidified 5% CO_2_/37 °C incubator. All experiments were performed on logarithmically growing cells. Cells were seeded at the specified density and allowed to attach overnight prior to adding compounds or reagents.

### 4.3. Cellular Viability Quantification

Cytotoxicity of bisPMB was evaluated using the standard MTT cellular viability assay. Briefly, WHCO1 cells were seeded at a density of 2.5 × 10^3^ cells per well in 90 µL in a 96-well culture dish. The following day 10 µL of bisPMB (0.5–25 µM) in DMSO (0.1% *v*/*v*) was added in quadruplicate to the cells and incubated for 24 h. Thereafter, 10 μL of 5 mg/mL 3-(4,5-dimethylthiazol-2-yl)-2,5-diphenyltetrazolium bromide (MTT; Sigma Aldrich, Saint Louis, MO, USA) was added and incubated with the cells for 4 h. The resulting formazan crystals were solubilised into 100 μL sodium lauryl sulphate (SLS) overnight at 37 °C and the absorbance at 595 nm was measured using a Multiskan FC multi-well reader (Thermo Scientific). The data was analysed using GraphPad Prism 4 software (GraphPad Inc., San Diego, CA, USA) fitted to a variable non-linear dose response curve from which the IC_50_ was obtained. 

### 4.4. Apoptosis Quantification

Apoptosis was detected using the Cell Death Detection Elisa PLUS kit (Roche Diagnostics, Rotkreuz, Switzerland) according to the manufacturer’s instruction. Briefly, WHCO1 cells at a density of 5 × 10^3^ cells per well were seeded in a 96-well culture dish and allowed to settle overnight. BisPMB in DMSO (0.1% *v*/*v*) was then added at the indicated concentration where ½ IC_50_ = 3.5 µM, IC_50_ = 7 µM and 2 IC_50_ = 14 µM and incubated with the cells for 24 h. Thereafter, cell lysates were prepared and the cytosolic fraction (20 µL) was transferred to a streptavidin coated plate for analysis. Briefly, the freshly prepared immunoreagent (80 µL) containing anti-histone biotin and anti-DNA linked peroxidase was added to each well and the plate was incubated on a shaker at room temperature for 2 h. The solution was then removed by gentle tapping, washed three times and then incubated with ABTS (100 µL) for 15 min. The reaction was quenched by adding the ABTS stop solution (100 µL) after which the absorbance at 405 nm and 495 nm was read using a Multiscan FC plate reader (Thermo Scientific).

### 4.5. Protein Synthesis Quantification

The effect of bisPMB on de novo protein synthesis was determined using the Click-iT HPG Alexa Fluor^®^ Protein Synthesis Assay Kit (Molecular Probes, Life Technologies) according to the manufacturer’s instruction. This assay tracks the incorporation of a labelled methionine analogue into proteins during active protein synthesis. This methionine amino acid is labelled with an alkyne group which becomes linked to a fluorescent azide via a click reaction at the end of protein synthesis. Briefly, 5 × 10^3^ WHCO1 cells were seeded into sterile white 96-well plates, allowed to adhere overnight and treated with bisPMB at the indicated concentration in DMSO (0.1 *v*/*v*) for 24 h. As control, cells were treated with the known protein synthesis inhibitor cycloheximide (Sigma-Aldrich) for 24 h at 3.5 µM concentration. Newly synthesised proteins were detected by fluorimetry using a Fluoroscan Ascent FL (Thermo Fisher Scientific) where E_ex_ 488 nm/E_em_ 538 nm. To normalise for cytotoxicity, parallel plates were treated identically and subjected to staining with 5 µM CFSE (Molecular Probes, Eugene, OR, USA) in PBS for 5 min at room temperature. After three washes with PBS cells were lysed in 100 µL 3% Triton X-100 and fluorescence was measured as stated above.

### 4.6. RNA Preparation and qRT-PCR

Total RNA was extracted from WHCO1 cells using the QIAzol® lysis reagent (QIAGEN, Venlo, Netherlands) and further purified with the RNeasy mini kit (QIAGEN) according the manufacturers’ instruction. Briefly, 1 × 10^6^ WHCO1 cells were seeded in 100 mm culture dishes for overnight attachment followed by treatment with ½ IC_50_ concentration of bisPMB for 24 h. RNA concentrations were measured using a Nanodrop 2000 (Thermo Fisher Scientific, Life Technologies) at an absorbance of 260 and 280 nm where an A260/280 ratio of 1.8–2.0 was considered pure. In all cases, RNA quality was assessed by 1.5% formaldehyde gel electrophoresis. In order to measure relative changes in gene transcription, quantitative polymerase chain reaction (*q*RT-PCR) was conducted using cDNA prepared from reversely transcribed RNA. Reverse transcription was performed with the ImpromII Reverse Transcription kit (Promega© Inc., Madison, WI, USA) on 3 µg total RNA according to the manufacturer’s instruction. Equal amounts (1 µL) of generated cDNA was then loaded into 96 well plates (Roche) in the presence of 2 × KAPA^TM^ SYBR® fast *q*RT-PCR master mix (KAPA Biosystems, Wilmington, MA, USA), 0.5 µmol forward and reverse primers (Table S2) and RNAse free water, totalling 20 µL per well, and run on a LightCycler® 480 Instrument II (Roche, Basel, Switzerland) under the following conditions: 5 min 95 °C, 35 × (10 s 95 °C, 20 s 55–61 °C, 10 s 72 °C). Gene expression was normalised to GAPDH using the 2^−ΔΔCt^ method [75]. 

### 4.7. Affymetrix Human Gene ST 2.0 Array and Bioinformatics Analysis

Total RNA was extracted in quadruplicate from control and bisPMB-treated WHCO1 cells as described above. The RNA quality was assessed by the RNA integrity number which was found to range from 9.9−10 in all cases using the Agilent 2100 Bioanalyser Eukaryotic Total RNA Nano Assay (Agilent Technologies, Santa Clara, CA, USA) with an internal control RNA sample of 20 ng/μL. The RNA was converted to biotin labelled complementary RNA (cRNA) which was then hybridized onto the Affymetrix Human Gene ST 2.0 array containing over 24,000 probe sets, and stained. The quantification of fluorescent intensity and distribution patterns of transcripts was obtained with the GeneChip® Scanner 3000 7G, from which the Affymetrix CEL files were generated. The Affymetrix CEL files containing the raw intensities of the probe set were imported onto the Partek® Genomics Suite™ software version 6.6 (Partek Inc., St Louis, MI, USA) and were assigned to categorical comparison groups based on the biological replicates of the samples. The samples were subsequently subjected to the robust multi array average (RMA) method, which was used for normalisation, background correction and summarisation of the probe intensity values. The 2 way analysis of variance (ANOVA) between the samples *p*-values was calculated to measure significances of differentially expressed genes (DEGs). These *p*-values were further corrected using the Bonferroni test for false discovery rate (FDR). Additionally, the cut-off for the significantly deregulated genes was set at −1.5 to +1.5 fold with an FDR *p*-value of α < 0.05. The DEG list generated using these parameters was then used for functional enrichment analysis. The ingenuity pathway analysis (IPA) software was used to generate significantly enriched canonical pathways and interactive gene networks http://www.ingenuity.com [76]. Gene Ontology analysis was performed using WebGestalt2 (WEB-based Gene SeT AnaLysis Toolkit) http://www.webgestalt.org.

### 4.8. Transient Knock Down of CHOP/GADD153 

In order to attenuate the expression of CHOP in WHCO1 cells, CHOP-specific small interfering RNA (siRNA) was used. Briefly, 1.5 × 10^5^ WHCO1 cells were seeded in a 12 well culture dish and allowed to settle overnight. Thereafter the cells were washed with PBS and transiently transfected with 40 nM CHOP siRNA (Sigma-Aldrich) or control siRNA (Santa Cruz Biotechnology, Dallas, TX, USA) using TransFectin™ Lipid reagent (Bio-Rad, Hercules, CA, USA) in serum free DMEM. Six hours post transfection the transfection media was removed and replaced with complete DMEM.

### 4.9. Immunoblot Analysis

For detection of proteins from WHCO1 cell lysates by immunoblot, standard protocols applied. Cells were seeded in 145 mm culture dishes at a density of 3 × 10^6^ cells and allowed to attach overnight. If kinase inhibitors were used (Sigma-Aldrich, Saint Louis, MO, USA), they were pre-incubated with the cells at the following concentrations: U0126 (1 µM, 5 µM, 10 µM) for 2 h or SP600125 (5 µM, 10 µM, 20 µM, 30 µM) for 30 min. Thereafter bisPMB in DMSO (0.1% *v*/*v*) at the indicated concentration or vehicle alone was added for the indicated time. Cells were then lysed using RIPA buffer (Cell Signalling Technology, Danvers, MA, USA) supplemented with proteinase inhibitor (Roche). Total protein was quantified using the Pierce® Bradford Protein Assay kit (Thermo Fisher Scientific, Life Technologies) according to the manufacturer’s instruction. Equal amounts (20–50 µg) of total protein lysates were separated by SDS-PAGE and transferred onto 0.2 µm nitrocellulose membranes (Bio-Rad) using conventional methods. After blocking with 5% non-fat milk the membranes were incubated with the following primary antibodies overnight at 4 °C: CHOP, JNK1/2, p-JNK1/2, p-ERK1/2 (Cell Signalling Technologies), GAPDH, Ubiquitin, ERK2 (Santa Cruz Biotechnology, Santa Curz, CA, USA). Specific antibodies were detected using appropriate horseradish peroxidase-conjugated secondary antibodies and were developed using the chemiluminescent reagent LumiGloReserve (KPL Incorporated, Delary Beach, FL, USA). A Page Ruler Plus Protein Ladder (Thermo Fisher Scientific, Life Technologies) was used to estimate molecular weight. Proteins were visualised using the UVP BioSpectrum™500 Imaging System (UVP, LCC, Upland, CA, USA), captured by the CCD camera (Canon Inc, Tokyo, Japan) and analysed with the Visionworks LS Acquisition analysis software (UVP).

### 4.10. Statistical Analysis

Student *t*-test, 1-way and 2-way ANOVA was used to ascertain the statistical significant differences between untreated and treated samples. *p* < 0.05 samples were considered significant where * *p*-value < 0.05; ** *p*-value < 0.01; *** *p*-value < 0.005; **** *p*-value < 0.001. 

## Supplementary Materials

The following are available online, Figure S1. Gene Ontology of biological processes ancestral categories in bisPMB-treated WHCO1 cells; Figure S2. Gene Ontology cellular component ancestral categories in bisPMB-treated WHC01 cells; Figure S3. KEGG Pathways enriched with bisPMB DEGs; Figure S4. KEGG pathway map showing significantly enriched ER protein processing pathway; Figure S5. Cellular compromise, cellular function and maintenance, cellular assembly and organization molecular gene network and functional categories for bisPMB; Table S1. Primer sequences of genes amplified by qRT-PCR, the amplicon size and the cycling conditions. The sequences were designed using the NCBI/Primer-BLAST; Table S2. The top 16 genes most deregulated by bisPMB in WHCO1 cells; Table S3.1. Gene Ontology of biological processes significantly enriched by bisPMB; Table S3.2. Gene Ontology of cellular components significantly enriched by bisPMB.
