# Supplementary material for: The Cytotoxicity of the Ajoene Analogue BisPMB in WHCO1 Oesophageal Cancer Cells Is Mediated by CHOP/GADD153
Source: Molecules. 2017 May 28;22(6):892. doi: 10.3390/molecules22060892 (PMC6152762; doi:10.3390/molecules22060892)
Supplement: Supplementary file 1 [file molecules-22-00892-s001.pdf]

Figure S1

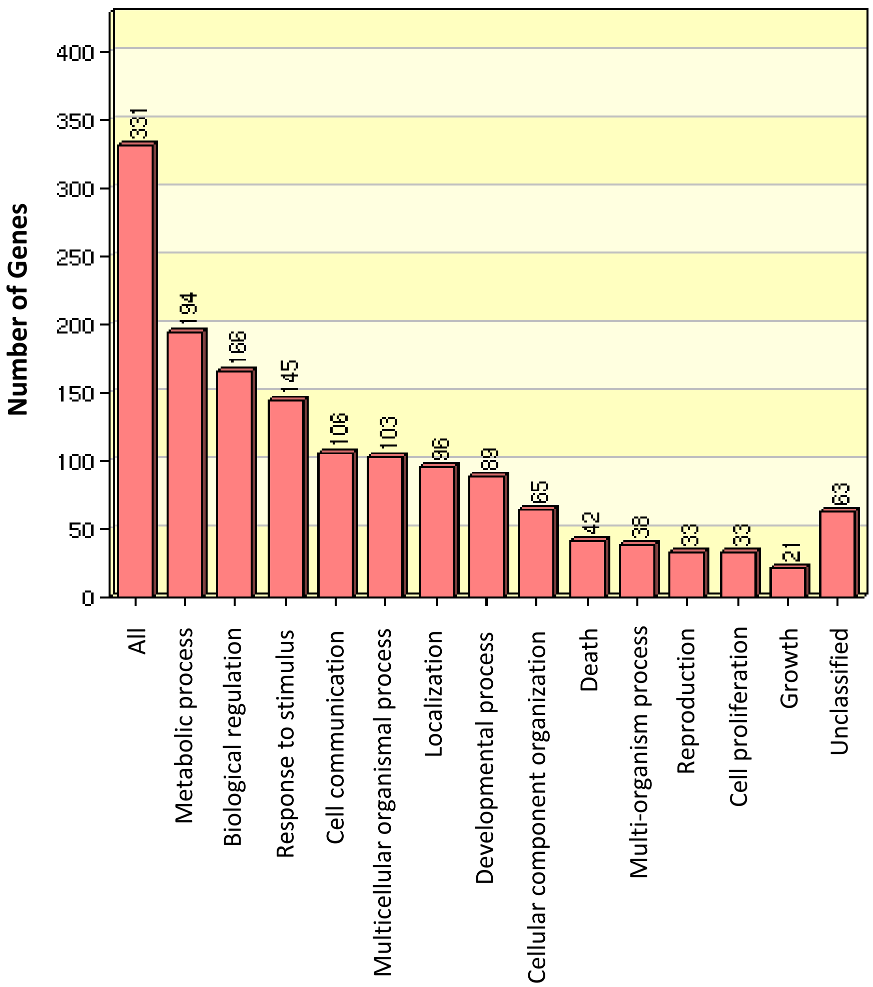

Figure S1. Gene Ontology of biological processes ancestral categories in bisPMB-treated WHCO1 cells. Each bar signifies broad ancestral biological process category and the numbers of bisPMB DEGs involved in each processes are displayed on top of each bar.

Figure S2

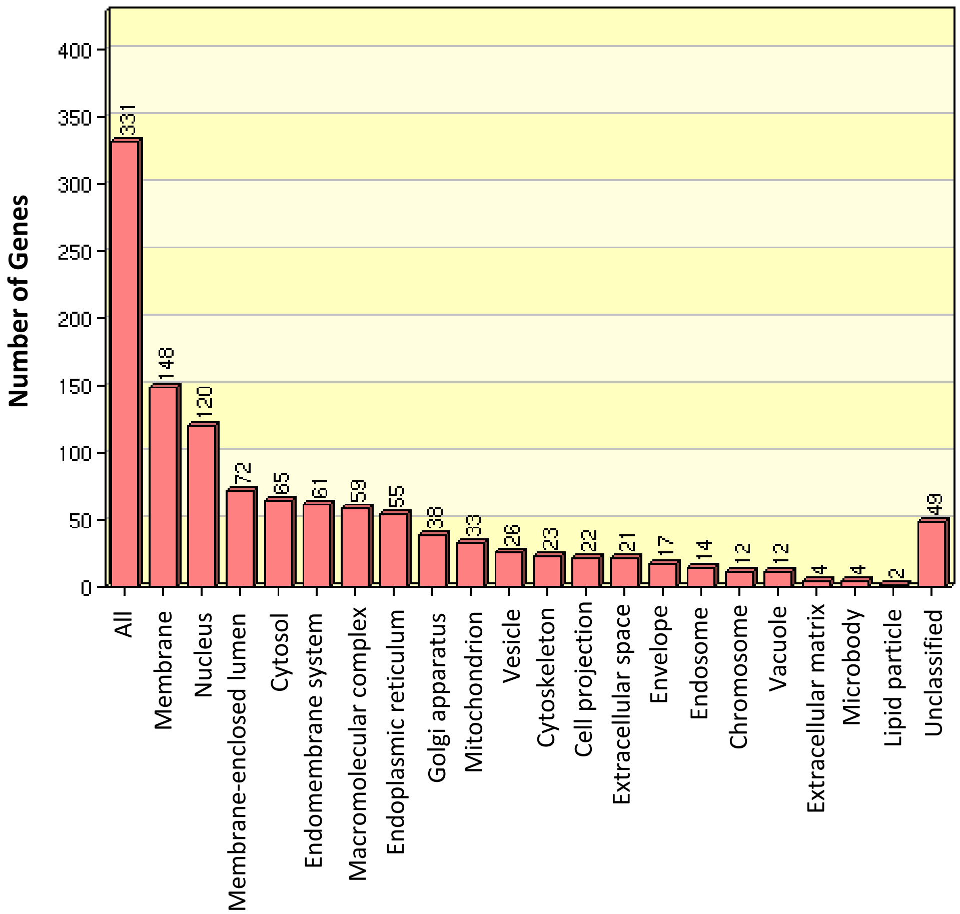

Figure S2. Gene Ontology cellular component ancestral categories in bisPMB-treated WHC01 cells. Each bar signifies broad ancestral cellular component and the numbers of bisPMB DEGs involved in each component are displayed on top of each bar.

Figure S3

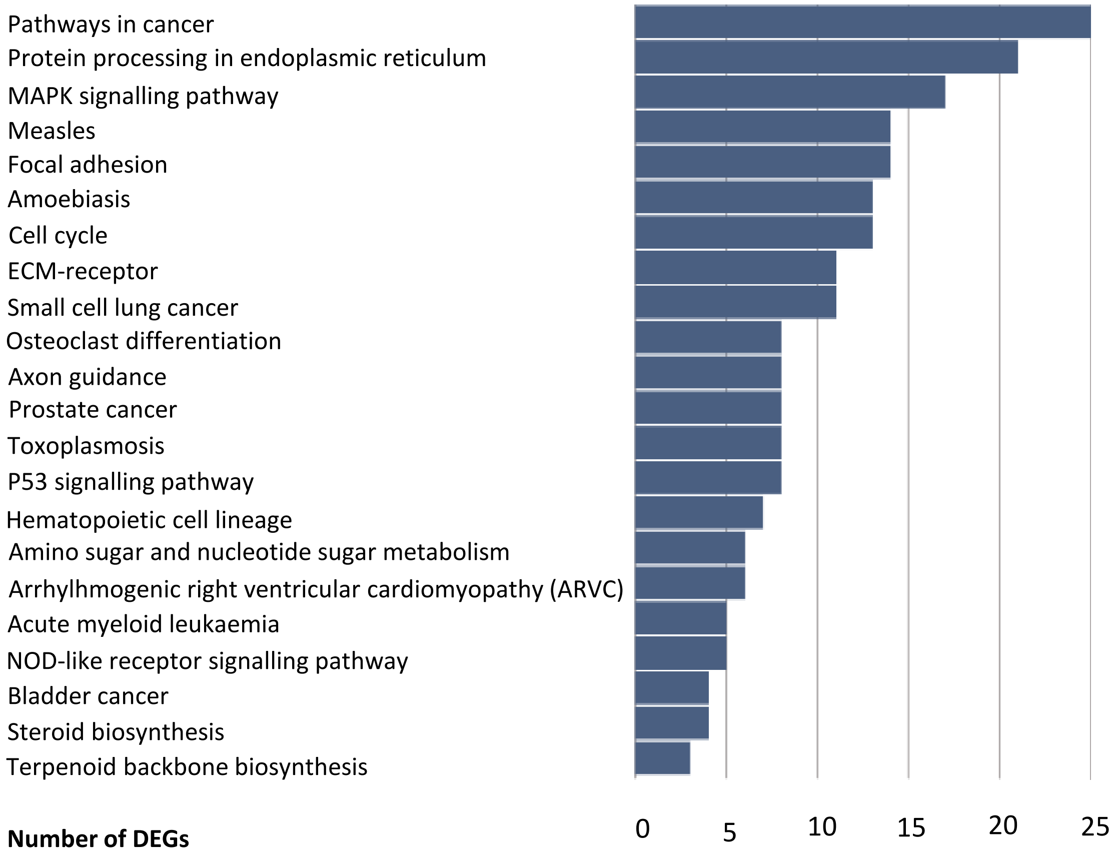

Figure S3. KEGG Pathways enriched with bisPMB DEGs. The bar chart indicates the number of DEGs represented in each pathway category. The names of the pathways are indicated on the y-axis and the number of DEGs present in each pathway is indicated on the x-axis.

Figure S4

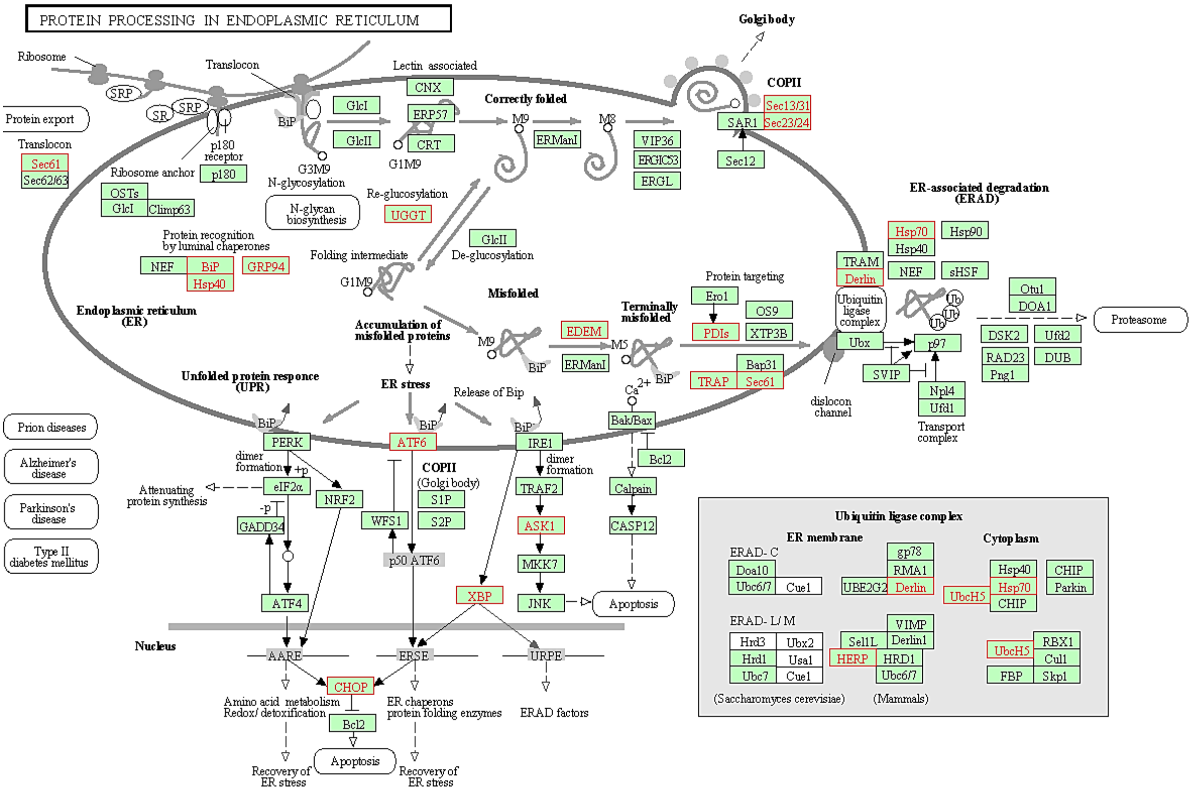

Figure S4. KEGG pathway map showing the significantly enriched ER protein processing pathway. The WebGestalt program was used to generate the significantly enriched KEGG pathways from a list of imported DEGs. Key: the green rectangular boxes represent gene products such as protein or RNA. The names of the gene products are written in black. The DEGs found in the bisPMB-treated cells are in red.

Figure S5

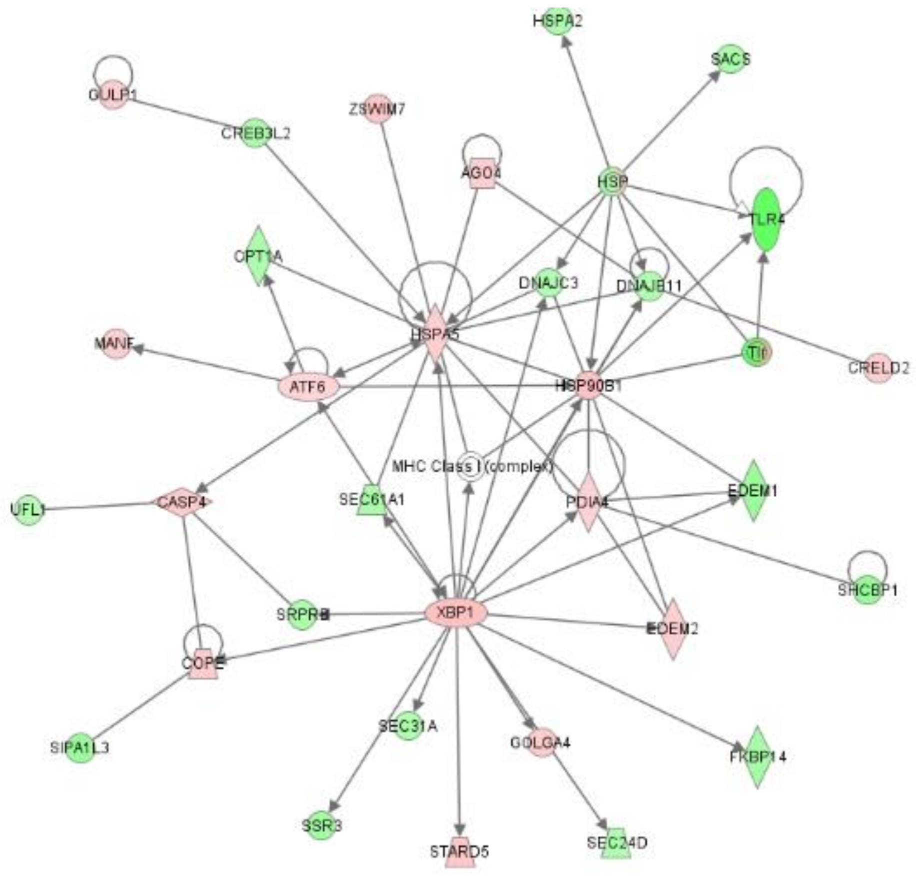

Figure S5. Cellular compromise, cellular function and maintenance, cellular assembly and organization molecular gene network and functional categories for bisPMB. The highest ranked gene network containing bisPMB DEGs. Green indicates down-regulated and red are up-regulated genes. The lines connecting the genes indicate the biological relationship between the genes.

Table S1. Primer sequences of genes amplified by *q*RT-PCR, with individual annealing temperatures and amplicon sizes. The sequences were designed using the NCBI/Primer-BLAST

Gene Symbol	*Forward Primer (5′ – 3′)*	*Reverse Primer (5′ – 3′)*	*Annealing T (°C)*	*Size (bp)*
GAPDH	GCCTGCTTCACCACCTTC	GGCTCTCCAGAACATCATCC	60	192
SEC63	TTCCAGTTGTTGTGGGCTCTT	CAGAAGCTCCAGCCAAAACC	60	153
RRBP1	GGCAGACCTCCTAGTGAAAG	GAGTTTTCGCCATCTCCTTGC	58	191
IIMAN1	CAGAATCCAAGATGGCGGGA	GGAATAGCATTCCCCGCGT	58	235
IRE1	GCCTAGTCAGTTCTGCGTCC	GCTGGTACTTCCAAAAATCCCG	60	184
SSR3	CTCAAGCACAAAGTAGCACAGA	GACCACGACCAGGAACAGAG	60	198
TRAM	GCGATTCGCAAGAAAAGCAC	TTGTTCTTCTGTTGCTGGGAGG	61	192
ERO1	GGTGCTTCTGCCAGGTTAGTG	GTCCCTTCTTCCACACTGGC	61	194
SEL1L	GAAACCAGCTTTGACCGCCA	AGCCCCACTTTTCATCTGCT	60	167
SEC61	ATTTCCAGGGCTTCCGAGTG	CGAGCTGAGAGCATTTGGGA	61	163
EDEM	TGTTCACAACAGAGGGACAC	CAGGTACACGATTGCAGTTGG	58	174
PDIA4	CACCAGAAGTCACGCTTGTGTTGA	AGGAGAACGCTTGCTGAGCTCCTT	60	161
ASK	CCCTGGAAACCCTGCATTTTG	GAGTCCGAGTTAGTATCACAG	57	196
ATF6	ACGGAGTATTTTGTCCGCCT	AGCAAAGAGAGCAGAATCCCAA	60	174
XBP1-S	TTACGAGAGAAAACTCATGGCC	GGGTCCAAGTTGTCCAGAATGC	55	283
GRP78	GACATCAAGTTCTTGCCGTT	CTCATAACATTTAGGCCAGC	50	260
CHOP	CCAGCAGAGGTCACAAGCAC	GGGAATGACCACTCTGTTTC	55	127

Table S2. The top 16 genes most deregulated by bisPMB in WHCO1 cells

UPREGULATED	fold	DOWNREGULATED	fold
TNFSF10	6.8	PTX3	-5.0
SERPINB4	6.2	ANKRD1	-3.2
KRT13	4.9	NTSR1	-3.1
SERPINB13	4.3	TMEM200A	-3.1
MMP12	3.9	ANTXR2	-2.9
PI3	2.0	MMP1	-2.2
SOX2	2.2	ADAM19	-2.1
IL8	2.3	TRBC2	-2.5

Table S3.1. Gene Ontology of biological processes significantly enriched by bisPMB. Bonferroni statistical test was used to calculate FDR (α<0.05).

**SIGNIFICANTLY ENRICHED BIOLOGICAL PROCESSES**	**DEGs**	**AdjP-value**
ER-nucleus signalling pathway	18	$9.03e^{-13}$
Response to topologically incorrect protein	21	$4.08e^{-13}$
Response to unfolded protein	21	$2.07e^{-13}$
Response to endoplasmic reticulum stress	20	$2.07e^{-13}$
Positive regulation of nuclease activity	15	$3.77e^{-12}$
Cellular response to topologically incorrect protein	17	$2.96e^{-12}$
Activation of signalling protein activity involved in unfolded protein response	15	$2.95e^{-12}$
Cellular response to unfolded protein	17	$1.48e^{-12}$
Endoplasmic reticulum unfolded protein response	17	$1.45e^{-12}$
Regulation of nuclease activity	15	1.35^{-11}

Table S3.2 Gene Ontology of cellular components significantly enriched by bisPMB. Bonferroni statistical test was used to calculate FDR (α<0.05).

**SIGNIFICANTLY ENRICHED CELLULAR COMPONENT**	**DEGs**	**AdjP-value**
Nuclear outer membrane-endoplasmic reticulum membrane network	34	$1.58e^{-07}$
Endoplasmic reticulum membrane	34	$1.21e^{-07}$
Endoplasmic reticulum	46	$1.12e^{-07}$
Cytoplasm	125	$5.00e^{-04}$
Endomembrane system	48	$1.00e^{-04}$
Intracellular	195	$1.18e^{-02}$
Intracellular membrane bound organelle	157	$1.18e^{-02}$
Endoplasmic reticulum lumen	9	$1.18e^{-02}$
